# Book Reviews

**Published:** 1870-09

**Authors:** 


### BIBLIOGRAPHICAL NOTICES.

*Manual of Discovery, Manufacture and Administration of Nitrous Oxide or Laughing Gas, in its Relation to Dental or Minor Surgical Operations, and Particularly for the Painless Extraction of Teeth.*—By F. R. Thomas, D.D.S. Published by S. S. White. of Philadelphia. This is a 12 mo. volume of 122 pages, the perusal of which will be productive of good results to all practicing dentists. It is a plain and practical manual on the manufacture and administration of Nitrous Oxide, and so condensed as to render it serviceable to every one in the profession. It is gotten up in the usual neat style of the publisher.

*Webster's Dictionary.*—Among our correspondence we find several letters of inquiry in regard to the best work of this kind for a library. The following is the opinion of the Hon. John L. Motley, the Historian, and now minister at the court of St James, concerning Webster's Unabridged Dictionary: "It has been, in common with other great lexicons of the English language, one of my daily companions. My testimonial to its erudition, the accuracy of its deffinitions, and to the vast etymological reseasch by which it has been enriched through the labors recently bestowed upon it, can hardly be of much value, sustained as the book is in world-wide reputation, by so general an approbation; I have no hesitation in expressing my sense of its merits."

*Transactions of the Sixth Annual Session of the Illinois State Dental Society.*—A volume of 116 pages recording the proceedings of the meeting held at Blomington, Illinois, May 10th, 1870.

